# Effects of Adding Various Silage Additives to Whole Corn Crops at Ensiling on Performance, Rumen Fermentation, and Serum Physiological Characteristics of Growing-Finishing Cattle

**DOI:** 10.3390/ani9090695

**Published:** 2019-09-17

**Authors:** Yawei Zhang, Xiangwei Zhao, Wanbao Chen, Zhenming Zhou, Qingxiang Meng, Hao Wu

**Affiliations:** 1 State Key Laboratory of Animal Nutrition, College of Animal Science and Technology, China Agricultural University, Beijing 100193, China; ywz718@163.com (Y.Z.); zhouzm@cau.edu.cn (Z.Z.); 2 Yishui Animal Husbandry Development and Promotion Center, Linyi 276400, China

**Keywords:** beef cattle, corn silage, complex lactic acid bacteria, growth performance, mixed organic acid salt

## Abstract

**Simple Summary:**

Silage additives, such as complex lactic acid bacteria inoculants and mixed organic acid salts, are effective at improving fermentation and preservation of corn silage. However, the effects of applying these additives at ensiling on beef cattle performance require further investigation. This study showed that corn silage, inoculated with complex lactic acid bacteria, significantly improved daily dry matter intake, ruminal ammonia nitrogen, and blood urea nitrogen; and numerically enhanced the average daily gain of growing–finishing bulls. Corn crops ensilaged with mixed organic acid salts alone or together with complex inoculants had no significant effect on animal performance, although it did alter some rumen fermentation characteristics and blood parameters. Our research contributes to the future development and selection of silage additives.

**Abstract:**

This study aimed to investigate the effect of applying various silage additives to whole corn crops at ensiling on growth performance, rumen fermentation, and blood physiology in growing–finishing bulls. Sixty Simmental × Yellow Cattle crossbred bulls were blocked by initial body weight (BW; 324.0 ± 5.4 kg) into 15 blocks. Animals in each block were randomly assigned to one of four diets formulated based on the following corn silage: control (CON), inoculated with complex lactic acid bacteria (CLB), ensilaged with mixed organic acid salts (MS), and ensilaged with CLB and MS (CLBMS). The feeding experiment lasted over 155 days, with an additional 7 days for adaptation. The results showed that bulls fed CLB-inoculated silage had greater (*p* < 0.05) daily dry matter intake than the other groups. The experimental treatment had no significant effect on average daily gain (*p* = 0.33) and feed-to-gain ratio (*p* = 0.13), although bulls fed CLB-inoculated silage had a larger numeric average daily gain. All additive-treated silage increased ruminal NH_3_–N content (*p* < 0.05) and reduced the acetate-to-propionate ratio (*p* < 0.05) of bulls compared with the control group. Bulls fed CLB-inoculated silage had a lower ruminal pH value (*p* < 0.05) than that of the other groups. Compared with the control group, bulls fed CLB-inoculated silage had greater blood cholesterol, albumin, and urea nitrogen (*p* < 0.05). Blood physiological responses were similar in bulls fed MS-treated and control silage, whereas those in cattle fed CLBMS-treated silage were between bulls fed CLB- and MS-treated silages and more similar to the former. Taking animal performance and cost effectiveness into consideration, the application of CLB alone to whole corn crops at ensiling appears to be a better choice compared with the application of either MS alone or both of them together.

## 1. Introduction

With the increase in global demand of meat and dairy products, particularly in developing countries such as China, the livestock sector is becoming one of the fastest-growing segments in the agricultural economy [1,2]. For ruminant production, an adequate and consistent supply of forage resources is a priority, due to their high proportion in the ration. Ensiling is an efficient strategy for moist forage feedstuff preservation, based on natural lactic acid fermentation under anaerobic conditions [3]. In recent decades, whole-plant corn silage has been become a popular choice for producers in areas where the land is suitable for cultivation because of its generally good ensiling characteristics, such as high biomass yield, relatively high metabolizable energy content, and a varying range of growth periods [4]. Although they form the basis of lactic acid fermentation, the presence of more highly fermentable substrates in both harvested crops and well-fermented silage make them highly susceptible to spoilage microorganisms [1] in the presence of oxygen during the silage processing and feed-out period, resulting in aerobic deterioration and nutrient loss [5]. Thus, a wide variety of silage additives have been suggested to assist in corn silage preservation [6].

Among common silage additives, complex lactic acid bacteria inoculants containing both homofermenters and heterofermenters and mixed organic acid salts, especially with potassium sorbate and sodium benzoate as the main active ingredients, have recently been used to enhance silage preservation [6]. Several studies have suggested that complex lactic acid bacteria inoculants can improve the aerobic stability of multiple types of silage, for instance, grass [7], whole-plant corn [8], and whole-crop barley [9]. Similarly, researchers have reported that both potassium sorbate and sodium benzoate are effective at improving the aerobic stability of silage [10,11] owing to their ability to inhibit the growth of yeast, which is commonly considered to be an initiator of aerobic deterioration [12]. However, to our knowledge, few studies have attempted to identify the effect of these additives on animal performance, especially beef cattle, when they are added to whole corn crops at ensiling, although improvements in animal performance are what producers expect while they add silage additives to plant material. Hence, in this study, a feeding experiment was conducted to investigate the difference in growth performance, serum physiology, and rumen fermentation of growing–finishing cattle fed diets formulated with whole-plant corn silage ensilaged with either complex lactic acid bacteria inoculants and mixed organic acid salt alones or together.

## 2. Materials and Methods 

The silage-making and feeding experiment was conducted at Longshengyuan Farm (118°72′ E, 35°62′ N, Shandong Province, China), an experimental demonstration station created to promote whole-plant corn silage by the Ministry of Agriculture of China, from October 2016 to June 2017. The animal study was approved by the China Agricultural University Animal Care and Use Committee (CAU2016-21176087). All the experimental procedures on animals described followed herein established guidelines for the care and handling of laboratory animals [13].

Whole plant corn was harvested at the maturation stage of half to two-thirds milk line and chopped to a theoretical particle length of 19 mm using a pull-type chopper (JAGUAR830, CLAAS KGaA mbH, Harsewinkel, German). Fresh harvested crops were randomly unloaded into four aboveground horizontal silos (100 × 8 × 3 m) with the following four treatments at ensiling, respectively: (1) No treatment as control (CON); (2) complex lactic acid bacteria (CLB)—2 g/t (fresh matter basis) of combination inoculants (SynLac I-HL, Synbio Tech Inc., Kaohsiung city, Taiwan) containing more than 1.0 × 10^11^ cfu/g of *Lactobacillus plantarum L28*, 1.0 × 10^9^ cfu/g of *Enterococcus faecium EF08*, and 1.0 × 10^9^ cfu/g of *Lactobacillus buchneri LBC136*; (3) mixed organic acid salts (MS)—100 g/t (fresh matter basis) of mixed organic acid salts, consisting of potassium sorbate (40%) and sodium benzoate (60%); (4) CLBMS: 2 g/t of CLB and 100 g/t of MS combined. These additives were dissolved into equivalent amounts of distilled water. The solutions were then evenly sprayed into the corresponding silos using an atomizer at a rate of 2 L/t (fresh matter basis); and equal amounts of water (2 L/t, fresh matter basis) were added to the corn plants in the remainder of the silos. The silage preservation method followed identical silage management practices, except for the additives, and lasted at least 42 days until feed-out. The fermentation characteristics and chemical composition of the four silage treatments are shown in Table 1.

A randomized block design was used for this animal experiment. A total of 60 *Simmental × Yellow Cattle* crossbred bulls (14-months old) with an initial body weight (BW) of 324.0 ± 5.4 kg (mean ± SEM), were blocked by weight into 15 blocks, with 4 animals per block. The animals in each block were randomly assigned to one of four experimental diets, formulated to meet the recommendation for growing and finishing cattle according to the Nutrient Requirements of Beef Cattle [14], based on the four treatments of whole-plant corn silage mentioned above: CON, CLB, MS, and CLBMS.

The experimental diet consisted of 60% of the respective corn silage, 16% corn grain, 6.8% corn husks, 7.0% wheat bran, 8.2% soybean meal, 1.0% limestone, 0.4% salt, 0.4% sodium bicarbonate, 0.1% of mineral premix (containing 8–15 g/kg of copper, 45–120 g/kg of ferrum, 25–65 g/kg of zinc, 15–60 g/kg of manganese, 250–1500 mg/kg of iodine, 100–700 mg/kg of cobalt, and 100–250 mg/kg of selenium), 0.01% vitamin premix (containing 30–85 million IU/kg of vitamin A, 7–38 million IU/kg of vitamin D3, and 150,000 IU/kg of vitamin E), and 0.01% monensin premix (containing 20% monensin) on a dry matter (DM) basis. The estimated chemical composition of the diets is shown in Table 2.

The bulls were housed in a series of individual pens (2 × 4 m) separated by an iron fence and equipped with an individual trough and drinking bowl. The total mixed rations were prepared from well-mixed concentrations and fresh corn silage using a fixed mixer (Ruiting Equipment Ltd., Zhengzhou, China), and then manually delivered to the corresponding animals with a proportionate residual of 10%. Water was available *ad libitum*. The feeding experiment lasted for 155 days, with an additional 7 days for adaptation. During the adaptation stage, the silage in the diets of the three treatment groups was replaced by the corresponding corn silage at once, while the silage in the diets of the control group remained unchanged. The diet during the adaption and measurement periods was the same. The daily amount of offered feed and refusals were manually measured using a weigh scale. Diets and silage samples were collected twice a month and stored at −20 °C for moisture and chemical composition analysis. The daily dry matter intake (DMI) of each bull was calculated as the difference between offered feed and refusals on a DM basis, and the average daily DMI of each bull over the whole experimental period served as the individual daily DMI. 

The live BW of each bull was weighed before feeding in the morning of two consecutive days at both the beginning and end of the experimental period; the average BWs were considered as the initial and final BW, respectively. Average daily gain (ADG) was calculated by subtracting the initial BW from the final BW and dividing the difference by the experimental duration of 155 days. The feed conversion ratio (FCR) was calculated by dividing DMI by BW gain during the experimental period. 

On days 90–92, since the beginning of the experiment, rumen fluid samples were collected individually with a syringe connected to an esophageal metal-coated rubber pipe (Type-K0021, ANSCITECH Ltd., Wuhan, China) within 1 h of feeding in the morning. For each animal, the first 100 mL of rumen fluid was discarded to avoid saliva contamination and the rest was filled into three 50 mL of centrifugal tubes. The pH of the rumen fluid was measured with a portable pH meter (Testo 205; Trsto AG, Schwarzwald, Germany). Rumen fluid samples were stored in liquid nitrogen until further analysis. At the same time, individual blood samples of all the bulls were collected with a vacuum tube by tail vein punctures in the morning after a 12-h fast, which were then centrifuged at 2500 × g and 4 ℃ for 15 min to separate the serum. The serum was snap-frozen using liquid nitrogen and stored at -80 ℃ until further analyses.

The silage and the other feed ingredients was analyzed for dry matter (DM, no. 934.01), crude protein (CP, no. 968.06), ether extract (EE, no. 920.39), ash (no. 942.05), and phosphorus (P, no. 965.17), according to methods recommended by the Association of Official Analytical Chemists (AOAC) [15]. Nitrogen concentration was determined with an elemental analyzer (Rapid N III, Elementar Analysensysteme GmbH, Langenselbold, Germany) and crude protein concentration was calculated as N × 6.25. Calcium concentration was determined using a flame atomic absorption spectrophotometer (WFX-320, BRAIC, Beijing, China) and phosphorus was measured with a UV spectrophotometer (UV-VIS 8500, Shanghai Tianmei Scientific Instrument Co., Ltd., Shanghai, China). The neutral detergent fiber (NDF) and acid detergent fiber (ADF) concentrations were determined using a method described by Van Soest et al. [16] in an ANKOM 2000 Fiber Analyzer (ANKOM Technologies, Macedon, NY) without sodium sulfite. Heat-stable α-amylase was used in the NDF assay.

To analyze the fermentation quality of the silage samples, silage slurry fluid was prepared from whole-plant corn silage by homogenizing 30 g fresh silage in 270 mL distilled water with a homogenizer (FLANK JT-C, Luohe Jintian Test Equipment Institute, Luohe, China), which was then centrifuged at 8000× g and 4 ℃ for 15 min. The supernatant was collected for later analyses. The volatile fatty acids (VFA) in both the silage extract and the rumen fluid samples were determined using a gas chromatograph (Type 3420, Agilent Tech Inc., Dionex, FTC, Palo Alto, USA), as described by Erwin et al. [17], and ammonia nitrogen was measured colorimetrically according to a method described by Broderick and Kang [18]. The lactic acid content in the silage extract was determined using an ion chromatograph (DIONEX-2500, Dionex, USA) equipped with a InoPac AS11-HC analytical column (4 mm × 250 mm), using a sodium hydroxide solution (50 mmol/L) as the mobile phase at a flow rate of 0.08 mL/min and temperature of 30 ℃. 

Regarding the serum physiological parameters, the activity of superoxide dismutase (SOD), alanine aminotransferase (ALT), aspartate aminotransferase (AST), alkaline phosphatase (ALP), creatine kinase (CK), lactate dehydrogenase (LDH), and the concentration of glucose (GLU), total protein (TP), albumin (ALB), cholesterol (CHO), creatinine (CRE), blood urea nitrogen (BUN), and total triglycerides (TG) was determined using a fully automated clinical chemistry analyzer (Selectral XL, ELITech Group, Puteaux, France) with commercial test kits (Biosino Bio-Technology and Science Incorporation, Beijing, China) following the manufacturer’s instructions. In addition, total antioxidative capacity (T-AOC), malondialdehyde (MDA), and catalase (CAT) were measured by an A6 Semi-Automated Bio-Chemistry Analyzer (Beijing Shiningsun Technology Co. Ltd., Beijing, China) with corresponding commercial test kits through a service corporation (Beijing Sino-UK Institute of Biological Technology, Beijing, China).

All data that were sorted preliminarily in Excel were imported into SAS (version 9.4, SAS Institute Inc., Cary, NC, USA) for further statistical analysis. Since this study adopted a randomized block design, a mixed model considering the additive treatment as a fixed effect and the weight block as a random effect was applied in the MIXED Procedure as follows:

Y_ij_ = µ + T_i_ + W_j_ + e_ij_ (1)

where Y_ij_ is the observation of bulls that belong to the j^th^ weight block and were offered a diet containing silage ensilaged with the i^th^ additive; µ and e_ij_ represent the general mean and random residual error, respectively; and T_i_ and W_j_ represent the effect of the additive-treated silage and the weight block, respectively. Differences in the least-squares means of the fixed effects were pairwise compared by a default approach (PDIFF) and declared significant at *p* < 0.05.

## 3. Results

The production performance of beef cattle fed diets containing whole-plant corn silage ensilaged with various additives are shown in Table 3. Additive treatments had a significant effect on DMI (*p* < 0.01), but not on the ADG (*p* = 0.33) or FCR (*p* = 0.13) of the animals. Bulls fed diets containing CLB- and CLBMS-treated silages had greater DMI (*p* < 0.05) than those fed MS-treated and untreated silages. Although there was no statistical difference (*p* > 0.05), animals fed a diet containing CLB-treated silage had maximal numeric ADG (1.67 kg/day), while this value was 1.54 kg/day for cattle fed CLBMS-treated silage, leading to a maximal numeric FCR for the latter group.

Rumen fermentation parameters in bulls fed various experimental diets are presented in Table 4. Silage treatments had a significant effect on the ammonia nitrogen content and pH of rumen fluid (*p* < 0.01). Cattle fed a diet containing CLB-treated silage had the greatest ammonia nitrogen content (67.02 mg/L) in the rumen, followed by animals fed diets containing CLBMS-treated (63.44 mg/L) and MS-treated (51.53 mg/L) silages. Bulls fed a diet containing untreated silage had the lowest ammonia nitrogen (21.84 mg/L) in the rumen. The pH of rumen fluid in cattle fed CLB-treated silage was 6.55, significantly lower (*p* < 0.05) than that in animals fed MS-treated, CLBMS-treated, and untreated silages, with values of 6.76, 6.71, and 6.76, respectively.

Although the treatments had no effect on total VFA (*p* = 0.25), animals fed a diet containing CLB-treated silage had a greater propionate content (*p* < 0.05) in their rumen, while cattle fed diets containing either MS- or CLBMS-treated silages had a greater iso-butyrate content (*p* < 0.05), compared with that in cattle fed untreated silage. In addition, butyrate content in the rumen of cattle fed a diet containing CLB-treated silage was greater (*p* < 0.05) than that of animals fed diets containing MS- and CLBMS-treated silages. Furthermore, the application of silage additives significantly lowered the ratio of acetate to propionate in the rumen of cattle, compared with that of bulls fed untreated silage (*p* < 0.05). Moreover, bulls fed a diet containing CLB-treated silage had a greater proportion of butyrate in total VFA, compared with that in cattle fed diets containing MS-treated and untreated silage (*p* < 0.05), while there was a lower iso-butyrate proportion in total VFA compared with that in animals fed the other diets (*p* < 0.05).

The serum physiological characteristics of bulls fed various experimental diets are summarized in Table 5. These parameters were classified into the following three groups based on their function: oxidative stability indicators, organ physiological indicators, and routine biochemical parameters. Adding silage additives to whole corn crops had no effect on antioxidant ability in the blood of animals (*p* > 0.05), except for the activity of SOD, which was greater (*p* < 0.05) in the blood of cattle fed CLBMS-treated silage than that of animals fed the other silages. Similarly, among organ physiological indicators, only the activity of CK was greater in the blood of bulls fed MS-treated silage than that in animals fed the other silages (*p* < 0.05). In addition, compared with cattle fed untreated silage, animals fed CLB-treated silage had greater CHO, ALB, and BUN (*p* < 0.05) in the blood, cattle fed MS-treated silage had greater CHO in the blood (*p* < 0.05), and bulls fed CLBMS-treated silage had greater GLU, TG, CHO, and BUN in the serum (*p* < 0.05).

## 4. Discussion

As reported [6,12], both homofermentative and facultative heterofermentative lactic acid bacteria can reduce proteolysis and fermentation loss by dominating in the early active fermentative period and suppressing undesirable microorganisms, while the obligate heterofermentative lactic acid bacteria (mainly *L. buchneri*) can improve the aerobic stability of silage by converting lactic acid to acetic acid after the active silage fermentation period. The development of complex lactic acid bacteria inoculants aims to simultaneously achieve the benefits of both types of inoculants, while ideally improving animal performance. The complex lactic acid bacteria inoculant used in the current study contains two strains of homofermenter (*L. plantarum L28 and E. faecium EF08*) and one strain of heterofermenter (*L. buchneri LBC136*). Dry matter intake was greater for bulls fed a diet containing CLB-inoculated silage than that for bulls fed a diet containing control silage, but the average daily gains and feed conversion ratio between cattle fed these two silages were not different. The greater nutrient intake of the diet containing CLB-inoculated silage by cattle might contribute to various factors, one of which is that the lactic acid bacteria originating from silage inoculants could survive in rumen and then interact positively with rumen microorganisms, altering rumen fermentation, increasing ruminal microbial biomass, and enhancing ruminal functionality [19,20]. In this study, cattle fed a diet containing CLB-inoculated silage had a lower pH; greater concentrations of ammonia nitrogen, propionate, and butyrate; and especially a lower ratio of acetate to propionate in the rumen, compared to bulls fed a diet containing control silage.

Few studies have investigated the effect of complex lactic acid bacteria inoculants on beef cattle production, with the exception of an animal study by Addah et al. [9]. The author compared a combination inoculant containing *L. buchneri*, *L. plantarum*, and *Lactobacillus casei* with an untreated control in whole-crop barley silage, finding that the feed intake for growing feedlot steers fed a diet containing the inoculated silage was less than that for steers fed a diet containing control silage. However, the average daily gains were similar between the two treatments, resulting in a higher feed efficiency for inoculated silage (0.19 vs. 0.17; *p* = 0.03). In another study, Ellis et al. [21] investigated the effect of silage treated with combination inoculants (mixture of *L. plantarum*, *Lactococcus lactis*, and *L. buchneri*) on the production performance of dairy cows. The results showed that dry matter intake, milk yield, and milk composition were not affected by silage inoculations. Differences in the strain of inoculant (*E. faecium EF08*, *L. casei or Lc. lactis*), silage material (corn, barley, or grass), and experimental animals (steers, bulls, or dairy cows) might account for the considerably disparate results between those studies and our experiment. In addition, in a study that examined the effect of feeding corn silage inoculated with combination inoculants (blends of *L. buchneri* and *L. plantarum*) on the growth performance of lambs, Basso et al. [22] found that the intakes of DM, CP, NDF, organic matter, and gross energy were improved in lambs fed inoculated silage, which agrees with our findings. More studies should be conducted to assess whether combination inoculants can consistently improve animal performance or production efficiency.

A greater concentration of ammonia nitrogen was found in the rumen of cattle fed CLB-inoculated silage than in bulls fed untreated silage (67.02 vs. 21.84 mg/L). A study of *in vitro* continuous culture fermentation with ruminal content indicated that the maintenance of ruminal fluid ammonia at 50 mg NH_3_–N/L is adequate to support maximal growth rates of rumen microorganisms [23]. Thus, in addition to less DMI, this could be attributed at least partly to more ammonia-utilizing microbes in the rumen of cattle fed untreated silage. Meanwhile, although the concentrations of total VFA, acetate, isobutyrate, valerate, and isovalerate in rumen fluid were similar between these two treatment groups, a greater concentration of propionate and butyrate would cause a lower pH and acetate-to-propionate ratio in the rumen of cattle fed CLB-inoculated silage. These results suggest that the rumen fermentation pattern was converted to propionic-type fermentation in cattle fed CLB-inoculated silage, compared with that in animals fed untreated silage, implying that the species and quantity of associated rumen bacteria might be different between these two groups. In short, the alteration of rumen fermentation between bulls fed CLB-inoculated and untreated silage indicates that the combination inoculants used in the current study might have interacted with rumen microbes to act as probiotics, as was found in previous studies [20,24].

Blood biochemical parameters have been commonly used to assess the plane of nutrition and health status in ruminants [25,26]. Among blood biochemical parameters, the concentrations of glucose, total triglycerides, creatinine, total protein, albumin, and blood urea nitrogen are usually used to evaluate the metabolic profile of carbohydrates, fats, and proteins, where it is considered that higher values mean better energy and protein nutrition when they vary within the normal range [27]. Albumin, blood urea nitrogen, and cholesterol are mainly synthesized in the liver [28], and greater concentrations of these metabolites in the blood of bulls fed CLB-inoculated silage were observed in this study, compared with that of cattle fed untreated silage, probably suggesting that the former had better hepatic metabolic activity and protein nutrition [28], which also accounts for the numerically higher average daily gain for bulls fed CLB-inoculated silage. Meanwhile, blood urea nitrogen is produced by the hepatic urea cycle from blood ammonia, which is absorbed in the gastrointestinal tract and is produced by rumen microbes from dietary degradable protein [27]. Thus, a greater concentration of blood urea nitrogen is also consistent with greater ammonia nitrogen content in the rumen and greater DMI for bulls fed CLB-inoculated silage, as discussed above.

Chemical additives with short-chain organic acids (as primary active antifungal ingredients) have been used to improve the aerobic stability of silages in some poor management situations, since they may be able to overcome the drawback of microbial inoculants, which take weeks to produce sufficient amounts of active-end products [29,30]. More recently, organic acid salts, for instance, potassium sorbate and sodium benzoate, have commonly been used in lieu of organic acids to improve the aerobic stability of silage [31,32,33], since they not only work by releasing the respective acid in silages, but are also less corrosive to machinery and less hazardous to workers [34]. Research has proved that sorbates and benzoates selectively inhibit specific yeasts and molds, while not affecting the growth of desirable bacteria such as lactic acid bacteria [35,36]. Meanwhile, potassium sorbate and sodium benzoate in acidic environments can also be inhibitory to some bacteria, such as clostridia [37] or spore-forming bacteria [36]. Thus, in theory, silage treated with blends of potassium sorbate and sodium benzoate appear to improve animal performance by increasing residual sugar content and decreasing proteolysis and aerobic deteriorative of silage. However, in the current study, we did not find any difference in growth performance (DMI, ADG, and FCR) between bulls fed MS-treated and untreated silage. In addition, there was little difference in fermentation quality or chemical composition between MS-treated and untreated silage (Table 1), as well as between corresponding experimental diets (Table 2). These results might be due to the low application rate of mixed organic acid salt additives in this study (100 g/ton). Indeed, many studies have proved that the effect of potassium sorbate and sodium benzoate on ethanol content, yeast counts, and aerobic stability of corn silage is dose-dependent [10,31,38].

In agreement with our findings, Huuskonen et al. [39] reported that timothy silage treated with mixed organic acid salts (active ingredients including sodium benzoate, potassium sorbate, and sodium nitrate) had no effect on the average dry matter intake of dairy bulls. Also, there was only a slight benefit from silage additives in animal performance, when silage was ensiled in round bales and its dry matter was 350–400 g/kg. Similarly, Nadeau et al. [40] reported that, compared with untreated grass silage, silage treated with mixed organic acid salts (active ingredients including potassium sorbate, sodium benzoate, sodium nitrite, and hexamine, applied at 2 L/ton) had no effect on silage fermentation characteristics, dry matter intake, and milk yield for dairy cows, although it decreased the urea content and somatic cell counts in milk. In a later experiment, when grass-clover silage treated with the same additive and untreated silage were fed to pregnant and lactating ewes and their lambs, Nadeau and Arnesson [41] found that there was no treatment effect on intake and body condition of the ewes. Lambs born from ewes fed treated silages had higher birth weights and live weight gains from birth to weaning than lambs from ewes fed untreated silage. However, the live weight gains for weaned lambs from weaning to slaughter was not affected by treatment, although greater DMIs were found for lambs fed treated silage.

Most of the rumen fermentation parameters were similar between cattle fed diet containing MS-treated and untreated silage. The explanation for the altered rumen fermentation by MS-treated silage is unclear, one of which might be that the activity of some rumen microbes was inhibited by the residual potassium sorbate and sodium benzoate in the well-fermented silage, considering the lack of significant difference in the nutritive value of either fermented silages (Table 1) or total mixed rations (Table 2) observed between these two treatments. Further, although the activity of creatine kinase and the concentration of cholesterol were greater in the blood of cattle fed MS-treated silage than in bulls fed untreated silage, the rest of the serum physiological parameters had no significant difference between these two treatments. Creatine kinase is a muscle-specific enzyme that increases with muscle catabolism, and cholesterol is a biomarker indicating hepatic metabolic activity [28]. However, because of individual differences, changes of one or two these biomarkers in the blood of animals are not an indicator of abnormal or impaired or enhanced organic function when there are no corresponding changes of the other measures [27]. Thus, the results in the current study were not enough to indicate a difference in the blood physiology between bulls fed MS-treated and untreated silage.

Similar to the animals fed CLB-inoculated silage, bulls fed CLBMS-treated silage had a greater DMI than that of animal fed untreated silage. However, there was no difference between the ADG of the cattle in these two groups, resulting in numerically lower feed efficiency (greater FCR) for bulls fed CLBMS-treated silage. In addition, rumen fermentation characteristics and serum physiological responses in bulls fed CLBMS-treated silage were in between bulls fed CLB-inoculated and MS-treated silage, but were more similar to the former. It is notable that bulls fed CLBMS-treated silage had a greater activity of superoxide dismutase. Superoxide dismutase is an important endogenous antioxidant enzyme that act as a component of first line defense system against reactive oxygen species [42]. Reactive oxygen species, especially super oxide anion radical, are constantly formed through a couple of normal cell reactions or processes, for instance, mitochondrial energy production pathway in normal body cell, and NADPH-Oxidase activity in white blood cells for destroying invading pathogens [42]. Therefore, the reason for increase in the plasma SOD enzyme activity in the bulls fed CLBMS-treated silage in this study could be due to increased expression of the enzymes as a compensatory mechanism in response to increased reactive oxygen levels [43]. From this point, a greater activity of superoxide dismutase might imply that bulls fed CLBMS-treated silage had a greater level of body metabolism, which might contribute to their lower growth performance. Overall, although the cause of the observations on bulls fed CLBMS-treated silage is unclear based on the current results, it seems likely that the application of mixed organic acid salts inhibited the activity of lactic acid bacteria in the combination inoculants when adding them together to the whole corn crops at ensiling, which then impaired the rumen fermentation, serum physiology, and even the animal performance. These results were not consistent with our expectations, and further research should be conducted to elucidate the cause of the negative outcome.

## 5. Conclusions

In summary, adding either complex lactic acid bacteria inoculants alone or together with mixed organic acid salts into whole corn crops at ensiling improved the dry matter intake of cattle, while CLB-inoculated silage improved the numeric average daily gains of the animals. All additive-treated silages altered rumen fermentation to a greater ammonia nitrogen and lower acetate-to-propionate ratio in the rumen of bulls, indicating that these treatments improved ruminal degradable protein and fermentation efficiency. Serum physiological responses in bulls fed CLB-treated silage were consistent with their ruminal fermentation and growth performance. Except for rumen fermentation parameters, MS-treated silage did not noticeably alter the production performance and serum physiological characteristics of cattle compared with untreated silage. CLBMS-treated silage appears to have a negative effect on growth performance, rumen fermentation, and serum physiological characteristics of cattle compared with CLB-inoculated silage. Overall, based on the present study and considering animal performance and cost effectiveness, adding complex lactic acid bacteria alone to whole corn plants at ensiling appears to be more efficient than applying mixed organic acid salts alone or in combination with complex lactic acid bacteria.

## Figures and Tables

**Table 1:** Fermentation parameters and chemical composition of corn silage ensilaged with various additives.

Item	Treatments ^1^			
	CON	CLB	MS	CLBMS
Fermentation parameters, mg/g DM				
Acetate	17.41	17.77	14.32	10.57
Propionate	1.04	0.73	0.85	0.71
Lactate	23.70	18.88	21.08	18.10
NH_3_–N	3.05	2.96	2.85	2.79
Nutritive values, %DM ^2^				
Dry matter	32.73	29.65	32.08	27.54
Crude protein	9.65	10.10	9.36	9.80
Soluble crude protein	4.74	5.85	5.07	4.35
Ether extract	4.49	3.42	4.97	4.20
Neutral detergent fiber	38.54	40.90	38.34	44.67
Acid detergent fiber	20.69	22.99	20.58	26.03
Ash	4.96	4.57	5.10	6.15
Starch	18.41	20.63	21.37	20.71
Metabolic energy, MJ/kg	10.79	10.38	10.88	10.50

^1^ CLB: corn silage ensilaged with complex lactic acid bacteria inoculants; MS: corn silage ensilaged with mixed organic acid salts; CLBMS: CLB and MS combined; CON: control. ^2^ ME was calculated based on the total digestible nutrients of each feed ingredient (ME = 0.82 × 4.409 × TDN) and their proportions, referring to NRBC (2016). The other nutrients are analyzed values.

**Table 2:** Estimation of chemical composition (% DM) and energy level of experimental diets.

Item ^1^	Treatments ^2^			
	CON	CLB	MS	CLBMS
DM	54.98	53.66	54.66	52.39
Crude protein	14.10	14.37	13.93	14.19
Ether extract	3.90	3.26	4.18	3.73
Neutral detergent fiber	31.64	33.06	31.53	35.32
Acid detergent fiber	15.10	16.49	15.04	18.31
Ash	5.52	5.28	5.60	6.23
Ca	0.73	0.73	0.72	0.74
P	0.31	0.35	0.31	0.35
ME, MJ/kg	11.22	10.96	11.26	11.05

^1^ ME was calculated based on the total digestible nutrients of each feed ingredient (ME = 0.82 × 4.409 × TDN) and their proportions, referring to NRBC (2016). The other nutrients are analyzed values. ^2^ CON: control; CLB: corn silage ensilaged with complex lactic acid bacteria inoculants; MS: corn silage ensilaged with mixed organic acid salts; CLBMS: CLB and MS combined.

**Table 3:** Growth performance of growing–finishing cattle fed diets containing whole-corn silage ensilaged with various additives ^1.^

Item ^2^	Treatments ^3^				SEM	*p*-Value
	CON	CLB	MS	CLBMS		
**DMI, kg/day**	11.29 ^b^	13.00 ^a^	11.37 ^b^	12.71 ^a^	0.21	<0.01
ADG, kg/day	1.53	1.67	1.57	1.54	0.03	0.33
FCR	7.57	7.87	7.30	8.35	0.16	0.13

^1^ Values with different superscripts within each row are significantly different (*p* < 0.05). ^2^ DMI: dry matter intake; ADG: average daily gain; FCR: feed conversion ratio (feed/gain); SEM: standard error of the mean. ^3^ CON: Control; CLB: corn silage ensilaged with complex lactic acid bacteria inoculants; MS: corn silage ensilaged with mixed organic acid salts; CLBMS: CLB and MS combined.

**Table 4:** Rumen fermentation parameters of growing-finishing cattle fed diets containing whole-plant corn silage ensilaged with various additives ^1^.

Item	Treatments ^2^				SEM	*p*-Value
	CON	CLB	MS	CLBMS		
NH_3_–N, mg/L	21.84 ^c^	67.02 ^a^	51.63 ^b^	63.44 ^ab^	3.16	<0.01
pH	6.76 ^a^	6.55 ^b^	6.76 ^a^	6.71 ^a^	0.02	<0.01
Total VFA, mmol/L	38.73	45.47	41.34	40.78	1.35	0.25
Individual VFA, mmol/L						
Acetate	27.45	29.95	28.25	27.55	0.80	0.60
Propionate	6.40 ^b^	8.98 ^a^	7.62 ^ab^	7.54 ^ab^	0.39	0.04
Isobutyrate	0.28 ^b^	0.43 ^ab^	0.54 ^a^	0.51 ^a^	0.03	0.02
Butyrate	3.74 ^b^	5.14 ^a^	4.03 ^b^	4.33 ^ab^	0.18	0.02
Isovalerate	0.82	0.93	0.89	0.82	0.03	0.34
Valerate	0.03	0.05	0.03	0.03	0.01	0.22
VFA profile, mol/100 mol						
Acetate	70.48 ^a^	66.16 ^c^	68.50 ^b^	67.89 ^bc^	0.35	<0.01
Propionate	16.52 ^b^	19.66 ^a^	18.22 ^ab^	18.13 ^ab^	0.33	0.01
Iso-butyrate	1.33 ^a^	0.64 ^b^	1.37 ^a^	1.14 ^a^	0.09	0.01
Butyrate	9.48 ^b^	11.41 ^a^	9.68 ^b^	10.72 ^ab^	0.24	0.02
Iso-valerate	2.12	2.03	2.17	2.06	0.05	0.81
Valerate	0.06	0.1	0.06	0.06	0.01	0.12
Acetate/Propionate	4.37^a^	3.43 ^b^	3.83 ^b^	3.79 ^b^	0.08	<0.01

^1^ Values with different superscripts within each row are significantly different (*p* < 0.05). ^2^ CON = Control; CLB: corn silage ensilaged with complex lactic acid bacteria inoculants; MS: corn silage ensilaged with mixed organic acid salts; CLBMS: CLB and MS combined.

**Table 5:** Serum physiological parameters of growing–finishing cattle fed diets containing whole-plant corn silage ensilaged with various additives ^1^.

Item	Treatments ^2^				SEM	*p*-Value
	CON	CLB	MS	CLBMS		
Antioxidant ability indicators						
Superoxide dismutase, U/mL	172.19 ^b^	176.47 ^b^	174.67 ^b^	193.61 ^a^	3.16	0.04
T-AOC, U/mL	11.04	10.91	11.92	9.32	0.59	0.49
Catalase, U/mL	60.81	58.82	55.49	57.78	2.11	0.87
Malondialdehyde, nmol/mL	5.10	3.96	4.21	4.20	0.30	0.56
Organ physiological indicators						
Alanine aminotransferase, U/L	26.45	22.66	22.70	24.28	0.68	0.13
Aspartate aminotransferase, U/L	76.4	64.10	62.49	64.19	2.53	0.14
Alkaline phosphatase, U/L	116.19	148.35	126.81	132.94	5.96	0.26
Lactate dehydrogenase, U/L	1306.69	1150.47	1174.66	1236.77	27.93	0.12
Creatine kinase, U/L	103.26 ^b^	116.41 ^b^	292.01 ^a^	156.06 ^b^	24.51	0.02
Cholesterol, mmol/L	2.25 ^b^	2.62 ^a^	2.61 ^a^	2.91 ^a^	0.07	<0.01
Routine biochemical parameters						
Glucose, mmol/L	4.31 ^b^	4.60 ^ab^	4.64 ^ab^	4.89 ^a^	0.06	<0.01
Total triglyceride, mmol/L	0.28 ^b^	0.29 ^ab^	0.28 ^b^	0.31 ^a^	0.01	0.11
Creatinine, µmol/L	148.42	150.12	140.48	155.14	2.63	0.25
Total protein, g/L	61.68	60.24	61.51	63.40	0.78	0.62
Albumin, g/L	27.51 ^b^	30.37 ^a^	28.00 ^b^	29.84 ^ab^	0.42	0.03
Blood urea nitrogen, mmol/L	2.89 ^b^	3.44 ^a^	2.69 ^b^	3.64 ^a^	0.09	<0.01

^1^ Values with different superscripts within each row are significantly different (*p* < 0.05); T-AOC: total antioxidative capacity. ^2^ CON: control; CLB: corn silage ensilaged with complex lactic acid bacteria inoculants; MS: corn silage ensilaged with mixed organic acid salts; CLBMS: CLB and MS combined.
